# Genetic Parameters for Methane Emissions Using Indirect Prediction of Methane and Its Association with Milk and Milk Composition Traits

**DOI:** 10.3390/ani12162073

**Published:** 2022-08-14

**Authors:** Heydar Ghiasi, Beata Sitkowska, Dariusz Piwczyński, Magdalena Kolenda

**Affiliations:** 1 Department of Animal Science, Faculty of Agricultural Science, Payame Noor University, Tehran P.O. Box 19395-4697, Iran; 2 Department of Animal Biotechnology and Genetics, Faculty of Animal Breeding and Biology, Bydgoszcz University of Science and Technology, 85-084 Bydgoszcz, Poland

**Keywords:** methane, lactose, fat, protein, heritability, correlation, Holstein-Friesian cows, prediction

## Abstract

**Simple Summary:**

Methane is a major source of greenhouse gases, and ruminant animals are mainly responsible for its emission. Measuring methane in a large group of animals is expensive and requires specialised equipment. Therefore, direct animal selection aiming at reducing methane production by dairy cows on a large scale is difficult. This study aims to predict methane production based on milk yield production, estimate heritability for methane production, and the genetic correlation between methane production and milk production. The indirect approach using milk traits and genetic parameters shows that methane production is a heritable trait. High genetic correlations were estimated between methane production and milk traits. That indicates the selection to decrease methane production would also negatively affect milk yield and composition.

**Abstract:**

The study covers milk yield and composition data for 17,468 Polish Holstein-Friesian cows. Methane production (g/lactation per cow, MP) for dairy cow were predicted using three methane production equations (MPE) that took into account: milk yield (MPE1), energy corrected milk (MPE2) and both milk protein concentration (%), and energy-corrected milk (MPE3). The average amounts of methane produced for each cow per lactation were 31,089 g, 46,487 g, and 51,768 g for MPE1, MPE2, and MPE3, respectively. Repeatability models were used to estimate genetic parameters for MP. The estimated heritabilities for MPE1, MPE2, and MPE3 were 0.30, 0.24, and 0.24, respectively, with a standard error of 0.01. High genetic correlations (>0.76) were obtained between methane and milk yield, protein, fat, lactose and dry matter contents in milk for MPE1, MPE2 and MPE3. Still, a moderate genetic correlation (0.34) was obtained between methane and fat content (MPE1); the standard error of the estimated genetic correlation was less than 0.05. The results of the current study indicate that genetic selection aimed to reduce MP in dairy cows is possible. However, such direct genetic selection could cause a negative genetic response in milk yield and composition due to negative genetic correlations between MP and milk yield and composition.

## 1. Introduction

Methane is a significant source of greenhouse gases, and ruminant animals are mainly responsible for methane emissions from anthropogenic sources [1]. Therefore, different ways are used to mitigate the methane production by dairy cattle farms. Such ways are the selection for higher productivity and efficiency, direct selection for reducing methane production, indirect selection based on correlated traits [2], and construction selection indices based on methane, milk production, and milk compositions [3]. Different methane phenotypes are recorded in the animal, such as methane production in grams or litres per day per cow. The main problem with using these traits (methane production in grams or litres per day per cow) in breeding programs is that these phenotypes are highly correlated with the amount of feed intake. When cows consume more feed, they produce more methane. Other phenotypes are methane intensity, defined as litres or grams of methane per kg of milk and methane yield, defined as grams or litters of methane per kg of dry matter intake [4]. The main problem with methane production and methane yield phenotypes is that measuring them in dairy cows requires more equipment. Additionally, the indirect prediction of these phenotypes is problematic because it needs to have data for feed intake that is not recorded on a large scale. On the other hand, methane intensity phenotype depends on milk yield, which can be used widely in indirect methane predictions. Several methods have been developed to measure methane production at the individual animal level, such as respiration chambers, the sulphur hexafluoride tracer technique, the in vitro gas production technique, the GrenFeed system, and the CO_2_ Technique [5]. However, measuring methane for a large number of animals is expensive and requires specialised equipment. Thus, the direct selection of methane emission traits for reducing methane production by a dairy cow on a large scale is difficult [6]. However, indirect features correlated with methane that can be easily and quickly recorded on a large scale could be considered. Methane production is correlated with dry matter intake, body weight, feed nutrient compositions, milk yield, and milk composition [7]. These traits are widely used to indirectly predict methane production in dairy cows [8,9,10]. More complex models (more input variables) for methane prediction will increase the prediction’s accuracy. Still, we should consider the trade-off between data availability and prediction accuracy [9].

For indirect methane production, milk production traits are easily measured and widely available in a commercial dairy production system. Milk fat is reported to be one of the main variables in developing an equation to predict methane production in dairy cows [11]. A moderate genetic correlation between methane yield, milk protein yield, and milk lactose yield has been reported [12,13]. Fatty acid profiles are other correlated milk composition traits readily available in dairy farms that could be used for methane prediction [14,15,16]. Biological pathways related to methane and fatty acids production in the rumen are common. Therefore, this trait can indirectly predict methane production [14]. Bittante et al. [17] reported the heritability of 0.19 to 0.27 for methane production using milk fatty acid profile as an indirect prediction of methane production. According to Breider et al. [18], maximisation in methane production and limiting associated effects in related traits, both methane production and MY, should be included as part of a selection index in national breeding goals. Extensive data to estimate breeding values are required to fit methane production into breeding goals. In most countries, direct measurements of methane production per cow are not available on a large scale. Therefore, in these countries, methane production is measured using a small number of cows and then predicted equations are developed to predict methane production on a large scale. Milk yield and composition are common traits used to create methane prediction equations.

This study aims to predict methane production based on milk yield and milk composition, estimate heritability for methane production, and estimate the genetic correlation between methane production and milk yield and milk composition.

## 2. Materials and Methods

### 2.1. Data

The herds selected for this study had more than 100 cows. The animals were characterised by high milk production (on average more than 10,000 kg of milk per lactation). The cows were kept in cowsheds throughout the year, in a free-standing system, with free access to the feed table. The differences were in the milking system. Milk yield and milk composition data (milk dry matter, milk protein, milk fat, and milk lactose yields; in total: 38,011 records) on 17,468 Polish Holstein-Friesian cows in parity 1 to 6 from 28 herds between 2007 and 2018 were used in the current study (Table 1).

### 2.2. Methane Production Equations

Three methane production (g/lactation per cow; MP) equations (MPE1–MPE3) that take into account milk yield (MPE1), energy-corrected milk (MPE2), and milk protein concentration (%) as well as energy-corrected milk (MPE3), developed by Niu et al. [9], were used (Table 2). The constructed predictive models were assessed using root-mean-square prediction error and concordance correlation coefficient.

### 2.3. Statistical Analysis

The model used for genetic parameters estimation was as follows:

y = Xb + Za + Wpe + e

where y is the vector of traits; b is the vector of fixed effect, containing parity (1–6), herd (1–28), year (1–16), the season of calving (1–4), and lactation length (200–400) as a covariate; a and pe are the vectors of random additive genetic and permanent environmental effects, respectively; e is the vector of residual effects; X is an = incidence matrix; and Z and W are design matrices. Genetic parameters were estimated using ASReml [19]. The model assumptions were as follows:

$$V(a)=A\sigma_{a}^{2},\;V(\mathrm{pe})=\;I_{n}\sigma_{pe}^{2},\;V(e)=I_{n}\sigma_{e}^{2},\;E(a)=0,\;E(\mathrm{pe})=0,\;\mathrm{Cov}(a,\mathrm{pe})=0$$

where $\sigma_{a}^{2}$,$\;\sigma_{pe}^{2}$ and $\sigma_{e}^{2}$ are additive genetic, permanent environmental, and residual variance, respectively. $I_{n}$ is the identity matrix of an order equal to the number of cows in the dataset. A is the numerator relationship matrix between animals (for which six generations of pedigree were used).

Heritability ($h^{2}$), repeatability (r), genetic correlation ( rg), and phenotypic correlation (rp) were calculated as follows:

$$h^{2}=\frac{\sigma_{a}^{2}}{\sigma_{P}^{2}}\;,\;r=\frac{\left({\;\sigma }_{pe}^{2}+\sigma_{a}^{2}\right)}{\sigma_{P}^{2}},\;\mathrm{rg}=\frac{CovG_{x,y}}{\sqrt{(\sigma_{gx}^{2}}\times \sigma_{gy}^{2})},\;\mathrm{rp}=\frac{CovP_{x,y}}{\sqrt{(\sigma_{px}^{2}}\times \sigma_{py}^{2})}$$

where $\sigma_{P}^{2}$ is phenotypic variance; and $CovG_{x,y}$ and $CovP_{x,y}$ are additive and phenotypic covariances between traits *x* and *y*, respectively.$\;\sigma_{gx}^{2}$,$\;\sigma_{px}^{2}$ are the additive genetic and phenotypic variances of trait *x* and $\sigma_{gy}^{2}$,$\;\sigma_{py}^{2}$ are the additive genetic and phenotypic variance of trait *y*.

## 3. Results and Discussion

The mean of methane production of Polish Holstein-Friesian cows per lactation predicted only by milk yield (MPE1) was 30,934 g, which was lower than the methane production predicted by the equation using ECM (MPE2) and milk protein concentration (%) and energy-corrected milk (MPE3). When we used ECM in MPE2 instead of milk yield, the mean of the predicted methane production increased by about 15,317 g. Adding protein yield and ECM in MPE3 increased the predicted methane production by about 20,571 g and 5254 g compared to MPE1 and MPE2, respectively. The average lactation length in the dataset used in our study was 310 days, with a range of 200–400 days. Dividing methane production (g/lactation per cow) by 310 yields methane production (g/day per cow) showed that the mean values for methane production/day/cow were 100, 149, and 166 g/day/cow in MPE1, MPE2, and MPE3, respectively. The mean value for predicted methane production/day/cow in the present study is significantly lower than in other reports in which direct methods of determining methane production were used. For example, the average methane production/day/cow in Holstein cows was reported as 331 g/day [20], 357 g/day [21], 315 g/day [22], 279 g/day [23], 831 g/day [4], and 350 g/day [24]. There are different methane prediction equations in the literature. Studies show that methane prediction equations, which include dry matter intake, metabolisable energy intake, fibre, and acid detergent fibre, are good for prediction compared to equations with only milk yield traits [9]. We used the methane prediction equation developed by Niu et al. [9], which is the most comprehensive equation as it uses more data and considers information from different regions of the world. Nevertheless, using an indirect prediction equation to predict methane production tends to be slightly biased. For example, Niu et al. [9] reported that using only milk yield and milk composition (milk fat and milk protein) tends to underpredict at the early and late stages of lactation.

### 3.1. Heritability and Genetic Correlations

The Akaike’s information criterion (AIC) of the models (Table 3) indicates that the MPE1 model is better than MPE2 and MPE3 for estimating the genetic parameters of methane production. Variance component, heritability, and repeatability of methane production in MPE1, MPE2, and MPE3 by single-trait analysis are presented in Table 3. Estimated variances were significant for MPE1, MPE2, and MPE3. The heritability estimated for methane based on MPE1 was 0.30, which was more significant than heritability in MPE2 and MPE3 (0.24). The repeatability of methane production for all models ranged from 0.40 to 0.45 (0.45 in MPE1 and 0.40 in MPE2 and MPE3). Both estimated repeatability and heritability in MPE2 and MPE3 were similar.

The values of the estimated heritability indicators were similar to those obtained by Lassen et al. [22] on Holstein cattle (0.21), Pszczola et al. [23] (0.27) and Sypniewski et al. [25] (0.22) on Polish Holstein-Friesian cattle, which used direct methods for measuring methane production. The obtained heritability for methane production indicates that genetic selection for decreasing methane production is possible.

Low heritabilities (0.12) for methane production in Holstein cows were reported by Saborío-Montero et al. [26]. Theirs was not in agreement with our obtained heritability. Van Engelen et al. [27] predicted methane production based on milk fat composition. They reported heritability between 0.12 and 0.44; thus, some estimates are in agreement and some are not with the heritabilities obtained in our study.

### 3.2. Genetic Correlations

For all models except for fat in MPE1, high positive genetic (0.76 to 0.96) and phenotypic (0.85 to 0.98) correlations were estimated between the predicted methane production with milk yield and milk composition (Table 4). The estimated genetic and phenotypic correlations in MPE2 were similar to those in MPE3. In the MPE1 model, which contains only milk yield, a moderate genetic correlation (0.34) was obtained between fat and methane production, while in other models (MPE2 and MPE3), these genetic correlations were high (0.85). In all models, the genetic correlation of methane production with milk yield was high. The highest values were estimated for milk yield (0.15) and fat yield (0.21). Breider et al. [18] also observed a positive correlation between methane production and milk yield. In the research of Calderón-Chagoyaet al. [3], the genetic correlation between the milk fat and protein percentages and methane emissions during milking was negative, −0.09 and −0.18, respectively. These contradictory results reported for genetic correlation between methane production, milk yield, and milk composition can be due to methane measurement methods and the accuracy of equations for methane production. Lassen and Løvendahl [22] estimated moderate genetic correlations (0.37 to 0.43) between methane emissions and milk yield. Breider et al. [7] reported a genetic correlation of methane production with milk yield ranging from 0.38 to 0.57 in different lactation weeks. These values were lower than the estimates obtained in the current study. It should be noted that the only source of information used in our research to predict methane production was milk yield and milk composition. Therefore, this can be one of the reasons that justify these high genetic correlations obtained between the predicted methane production and milk yield and its composition. A high genetic correlation between lactation milk yield and the predicted methane production is expected because a high milk production needs a high dry matter intake, resulting in high methane production.

The estimated genetic correlations between milk protein and predicted methane production were high, 0.82 in MPE1 and 0.76 in MPE2 and MPE3 (Table 4). Kandel et al. [12] used different equations to predict methane production in dairy cows and obtained lower genetic correlations between methane and milk protein (0.14–0.38) than our estimates. These estimates are not in agreement with the result reported by Bittante and Cecchinato [17], which estimated the genetic correlation between the protein (percentage) in milk with methane production to be −0.23. Lassen and Løvendahl [22] reported low to moderate positive genetic correlations (0.15–0.37) of methane production with fat- and protein-corrected milk. In our study, high genetic correlations were obtained between methane with milk lactose, milk fat, and milk dry matter, except in MPE1, where a moderate (0.34) genetic correlation was obtained between methane production and milk fat. Kandel et al. [28] predicted methane emissions from milk mid-infrared spectra. They reported a positive weak genetic correlation (0.11 to 0.12) between methane production and milk fat yield and a negative (−0.04 to −0.05) one between methane production and milk protein yields. A low genetic correlation (0.19) between methane production and milk lactose yield was reported by Deharenget al. [29], which was not in agreement with the genetic correlations (0.75 and 0.98) obtained in our study. This shows that the genetic correlations obtained in our study agree with some studies and not others. This can be due to the different phenotype measurements for farm methane production and feeding strategies. Kandel et al. [28] suggested that the negative genetic correlation of methane production with milk yield might have been caused by high-performance cows being fed with a high amount of concentrates, which caused cows to produce less methane than cows fed with a high amount of roughage. Therefore, the feeding strategy in herds used in our study can affect the high genetic correlation. The trait we defined for methane measuring in the current study was g/lactation/cow, which was a linear trait. Some methane production measures were ratio traits (methane yield kg/kg of dry matter intake; methane intensity kg/kg of output). However, our method of phenotypic measurement of methane can show the high genetic correlation between methane and milk, milk fat, milk protein, and milk dry matter. However, according to these obtained correlations, it may be concluded that animals with a high genetic potential for milk production will also have a high genetic potential for methane production [22]. Kirchgessner et al. [30] demonstrated that genetic improvements in milk production would increase the total amount of methane production per animal per day; however, the amount of methane production per kg of milk (methane intensity) would be reduced. The genetic correlations obtained between methane and milk, milk fat, milk protein, and milk dry matter in our study can be explained by the fact that high-producing cows require more energy. Cows that consume more energy produce more methane, although the production level depends on the consumption of concentrates or roughage [12]. According to the results obtained in our study, direct selection to reduce methane production may result in a lower milk yield and lower content of fat, protein, and lactose due to the high genetic correlation estimated between methane production and milk yield and milk compositions.

## 4. Conclusions

The indirect approach using milk traits and genetic parameters shows that methane production is a heritable trait. High genetic correlations were estimated between predicted methane production and milk traits. The results of the current study indicate that the selection aiming at decreasing methane production may also negatively affect milk yield and composition.

## Figures and Tables

**Table 1:** Summary statistics of the analysed data.

Variable	Mean	Standard Deviation	Maximum	Minimum	Number of Cows	Number of Records
Milk yield (kg/lactation)	11,221	2353	18,495	6001		
Fat content (kg/lactation)	428	92	714	152	17,468	38,011
Protein content (kg/lactation)	375	74	608	175		
Lactose content (kg/lactation)	546	114	899	257	17,468	38,011
Milk dry matter (kg/lactation)	1431	284	2330	694		
Methane1 ^a^ (g/lactation per cow)	30,934	6426	50,790	16,682	17,468	38,011
Methane2 ^b^ (g/lactation per cow)	46,251	9170	75,425	21,917		
Methane3 ^c^ (g/lactation per cow)	51,505	10,239	84,080	24,334	17,468	38,011

^a^ Indirect prediction of methane production using milk yield; ^b^ indirect prediction of methane production using energy-corrected milk; and ^c^ indirect prediction of methane production using energy-corrected milk and milk protein.

**Table 2:** Methane production (g/lactation per cow) equations (MPE).

Equation	Prediction Equation ^a^	Models Performance	
		RMSPE ^b^, %	CCC
MPE1	299 (12.1) + 2.73 (0.171) × MY	21.7	0.51
MPE2	259 (11.1) + 3.86 (0.167) × ECM	20.3	0.59
MPE3	150 (16.1) + 4.31(0.172) × ECM + 28.3 (3.20) × CPC	19.8	0.62

^a^ Numbers in the brackets are the standard errors of estimated intercepts and slopes of equations; MY, milk yield (kg/lactation); ECM, energy-corrected milk (kg/lactation) that is calculated as ECM (kg/lactation) = (0.327 × milk (kg/lactation)) + (12.95 × fat yield (kg/lactation)) + (7.2 × protein yield (kg/lactation)); CPC, milk protein concentration (%). ^b^ RMSPE, root-mean-square prediction error; CCC, concordance correlation coefficient.

**Table 3:** Estimated genetic variance of methane and Akaike’s information criterion (AIC) for each methane prediction equation (MPE).

MPE	Variance Component			Heritability (Standard Error)	Repeatability (Standard Error)	AIC
	Additive	Permanent	Residual			
MPE1	6,771,940	3,371,540	12,418,000	0.30 (0.01)	0.45 (0.01)	680,482
MPE2	10,345,900	6,870,700	25,606,200	024 (0.01)	0.40 (0.01)	706,754
MPE3	12,898,700	8,566,120	31,924,400	024 (0.01)	0.40 (0.01)	715,148

**Table 4:** Phenotypic (rp) and genetic (rg) correlations of predicted methane with milk and milk composition traits in each methane prediction equation (MPE).

**MPE**	**Milk**		Protein		Fat		Lactose		Milk Dry Matter	
	rp	rg	rp	rg	rp	rg	rp	rg	rp	rg
MPE1	0.94	0.82	0.92	0.82	0.66	0.34	0.92	0.98	0.94	0.88
MPE2	0.89	0.76	0.92	0.85	0.82	0.85	0.88	0.75	0.98	0.96
MPE3	0.89	0.76	0.92	0.85	0.92	0.85	0.88	0.75	0.98	0.96

The standard error of all correlations was less than 0.03.

## Data Availability

The data presented in this study are available on request from the corresponding author.
